# Repression of Rgg But Not Upregulation of LacD.1 in *emm1*-type *covS* Mutant Mediates the SpeB Repression in Group A *Streptococcus*

**DOI:** 10.3389/fmicb.2016.01935

**Published:** 2016-11-29

**Authors:** Chuan Chiang-Ni, Teng-Ping Chu, Jiunn-Jong Wu, Cheng-Hsun Chiu

**Affiliations:** ^1^Department of Microbiology and Immunology, College of Medicine, Chang Gung UniversityTao-yuan, Taiwan; ^2^Graduate Institute of Biomedical Sciences, College of Medicine, Chang Gung UniversityTao-Yuan, Taiwan; ^3^Molecular Infectious Disease Research Center, Chang Gung Memorial HospitalTao-yuan, Taiwan; ^4^Department of Medical Laboratory Science and Biotechnology, College of Medicine, National Cheng Kung UniversityTainan, Taiwan; ^5^Department of Biotechnology and Laboratory Science in Medicine, School of Biomedical Science and Engineering, National Yang-Ming UniversityTaipei, Taiwan; ^6^Department of Pediatrics, Chang Gung Children’s HospitalTao-yuan, Taiwan

**Keywords:** group A *Streptococcus*, CovR/CovS, Rgg, LacD.1, SpeB

## Abstract

CovR/CovS is an important two-component regulatory system in human pathogen group A *Streptococcus* (GAS). Epidemiological studies have shown that inactivation of the sensor kinase CovS is correlated with invasive clinical manifestations. The phosphorylation level of response regulator CovR decreases dramatically in the absence of CovS, resulting in the derepression of virulence factor expression and an increase in bacterial invasiveness. Streptococcal pyrogenic exotoxin B (SpeB) is a cysteine protease and is negatively regulated by CovR; however, the expression of SpeB is almost completely repressed in the *covS* mutant. The present study found that in the *emm1-*type A20 strain, non-phosphorylated CovR acts as a transcriptional repressor for SpeB-positive regulator Rgg. In addition, the expression of Rgg-negative regulator LacD.1 is upregulated in the *covS* mutant. These results suggest that inactivation of Rgg in the *covS* mutant would directly mediate *speB* repression. The current study showed that overexpression of *rgg* but not inactivation of *lacD.1* in the *covS* mutant partially restores *speB* expression, indicating that only *rgg* repression, but not *lacD.1* upregulation, contributes to the *speB* repression in the *covS* mutant.

## Introduction

*Streptococcus pyogenes* (group A *Streptococcus*, GAS) is an important human pathogen causing diseases including pharyngitis, tonsillitis, scarlet fever, cellulitis, necrotizing fasciitis, and toxic shock syndrome. There are more than 500,000 deaths each year due to GAS-related diseases (Carapetis et al., 2005), indicating that invasive GAS infections are still a major clinical problem around the world. Mutations in the *covS* gene are detected more frequently in clinical isolates from severe invasive infections than in non-invasive isolates (Sumby et al., 2006; Ato et al., 2008; Ikebe et al., 2010; Lin et al., 2014; Friaes et al., 2015). CovS is the sensor kinase that phosphorylates an aspartic residue (D53) of the response regulator CovR (Levin and Wessels, 1998; Horstmann et al., 2014, 2015). Acquisition of null *covS* alleles causes a repression of streptococcal pyrogenic exotoxin B (SpeB) and upregulation of many virulence factor-encoding genes, resulting in increased GAS virulence during infection (Sumby et al., 2006; Walker et al., 2007; Ikebe et al., 2010; Friaes et al., 2015).

Streptococcal pyrogenic exotoxin B is a secreted cysteine protease of GAS (Chiang-Ni and Wu, 2008; Olsen and Musser, 2010). A DNase I footprint assay showed that recombinant CovR binds to the -53 to +36 region of the *speB* promoter (+1, transcription start site) (Miller et al., 2001). In addition, phosphorylation of CovR further enhances its binding to the *speB* DNA probe (Miller et al., 2001). Furthermore, deletion of the *covR* gene results in an increase in *speB* expression (Heath et al., 1999; Miller et al., 2001; Graham et al., 2002), indicating that CovR acts as a transcriptional repressor of *speB*. However, expression of *speB* is almost completely repressed in the *covS* mutant (Sumby et al., 2006; Walker et al., 2007; Trevino et al., 2009; Tran-Winkler et al., 2011). Transcriptome analysis showed that expression of the SpeB positive regulator Rgg is decreased accordingly in the *covS* mutant compared with that in the wild type strain (Sumby et al., 2006), suggesting that the decrease in *speB* transcription may be caused by the repression of *rgg*. However, the role of Rgg in the regulation of *speB* expression in the *covS* mutant has not been clearly demonstrated.

The location of *rgg* is adjacent to that of the *speB* gene in GAS chromosome. Rgg protein binds to the *speB* promoter specifically and is essential for the transcription of *speB* (Neely et al., 2003). Expression of both *rgg* and *speB* is only detected in late-exponential to stationary phases of growth (Unnikrishnan et al., 1999; Neely et al., 2003). However, expression of *rgg* from a promoter expressed during the early-log phase of growth cannot trigger *speB* expression, indicating that the regulatory activity of Rgg is controlled by other regulatory molecules. (Neely et al., 2003). Loughman and Caparon (2006) showed that Rgg activity is regulated by an aldolase-like protein, LacD.1. On NaCl supplementation and at neutral pH conditions, LacD.1 could bind to Rgg protein to restrict its activation of *speB* transcription (Loughman and Caparon, 2006). In addition, Anbalagan et al. (2012) showed that DNA-binding specificity of Rgg is responsive to environmental changes in a LacD.1-dependent manner. Rgg coordinates virulence factor expression, catabolic activity, and thermal and oxidative stress responses (Chaussee, 2002; Chaussee et al., 2003, 2004; Pulliainen et al., 2008). Therefore, interactions between LacD.1 and Rgg could be important in the pathogen’s virulence and adaptation to environmental changes during infection.

The present study found that expression of *lacD.1* was significantly increased in the *covS* mutant compared with that in the wild type strain. Therefore, our aim was to elucidate the role of the Rgg-LacD.1 system in *speB* repression in the *covS* mutant. The results indicate that only *rgg* repression, but not the upregulation of *lacD.1*, contributes directly to *speB* inhibition in the *covS* mutant.

## Materials and Methods

### Bacterial Strains and Culture Conditions

Group A *Streptococcus* strain A20 (wild type strain) is an *emm1*-type strain and has been previously described (Chiang-Ni et al., 2009). Strain AP3 was provided by Prof. Jiunn Jong Wu (Department of Medical Laboratory Science and Biotechnology, College of Medicine, National Cheng Kung University, Tainan, Taiwan). This strain was isolated from the spleen of A20-infected BALB/c mouse (subcutaneous infection) after 3 days of infection. In this study, the genome sequence of AP3 was generated using the Miseq sequencer (Illumina) according to the manufacturer’s protocols. Sequencing reads were mapped; and SNPs and Indels were called relative to the A20 genome sequence (NCBI accession number: NC_018936). The 143T deletion in the *covS* gene was found and confirmed by traditional Sanger sequencing method. In addition, another six SNPs and an Indel were found in the repeat sequence regions of transposases or rRNA genes (data not shown). GAS strains were cultured on the trypticase soy agar with 5% sheep blood or in the tryptic soy broth (Becton, Dickinson and Company, Sparks, MD) supplemented with 0.5% yeast extract (TSBY) and described in **Table 1**. *E. coli* DH5α was purchased from Yeastern (Yeastern Biotech Co., LTD, Taipei, Taiwan) and was cultured in Luria-Bertani (LB) broth at 37°C with vigorous aeration. When appropriate, antibiotics chloramphenicol (25 and 3 μg/ml for *E. coli* and GAS, respectively), erythromycin (125 and 5 μg/ml for *E. coli* and GAS, respectively) and spectinomycin (100 μg/ml) were used for selection.

**Table 1:** Group A *Streptococcus* (GAS) strains used in this study.

Strain	Parental strain	Description	Reference
A20	-	*emm1*/ST28 wild type strain	Chiang-Ni et al., 2009
AP3	A20	A20 animal-passage strain with early translational termination mutation in the *covS* gene	This study
SW656	A20	*covR* isogenic mutant	This study
SW934	AP3	*covR* isogenic mutant	This study
SCN121	AP3	*covR/covS trans*-complementation strain	This study
SCN127	AP3	pTRKL2 vector control strain	This study
SCN128	A20	CovR_D53A_ mutation strain	This study
SCN134	AP3	*rgg* overexpression strain (pDL278-*rgg*)	This study
SCN139	A20	*lacD.1* isogenic mutant	This study
SCN140	AP3	*lacD.1* isogenic mutant	This study
SCN141	SCN128	*lacD.1* isogenic mutant	This study


To treat GAS strains with neutral and acidic broths, bacterial pellets (collected from cultures in 40 ml of TSBY for 12-16 h at 37°C) were washed and resuspended in 1 ml of fresh TSBY broth. Seventy-five microliter of bacterial suspension was transferred to TSBY broth that was adjusted to pH 7.5 (supplemented with 0.1 M of HEPES) or pH 6.0 (supplemented with 0.1 M of MES) by 1 N HCl or NaOH (Dalton and Scott, 2004). Although culture broths were buffered by salts, pH of bacterial suspensions was decreased significantly after 4-5 h of incubation. Therefore, GAS strains were cultured at different pH conditions for 3 h at 37°C. The O.D._600_ of the bacterial suspensions (both pH 7.5 and pH 6.0) after 3 h incubation were around 0.4-0.6.

### DNA and RNA Manipulations

Group A *Streptococcus* genomic DNA extraction, RNA extraction, and reverse transcription were performed as described previously (Chiang-Ni et al., 2009). Real-time PCR reactions were performed in a 20 μl mixture containing 1 μl of cDNA, 0.8 μl of primers (10 μM), and 10 μl of SensiFAST^TM^ SYBR Lo-ROX pre-mixture (Bioline Ltd, London, UK) according to the manual. The expression level of the target gene was normalized to the *gyrA* and analyzed using the ΔΔCt method (7500 software v2.0.5, Applied Biosystem^®^, Thermo Fisher Scientific Inc.). In addition, all values of control and experiment groups were divided by the mean of control samples before statistical analysis (Valcu and Valcu, 2011). Primers used for real-time PCR analysis were designed by Primer3^1^ (v.0.4.0) according to the MGAS5005 sequence (NCBI reference sequence: NC_007297.1) and are described in **Table 2**. Southern blot and Northern blot analyses were performed as described previously (Chiang-Ni et al., 2012). Ten microgram of genomic DNAs extracted from GAS strains were digested by restriction enzymes for Southern blot analysis. Three microgram of RNAs extracted from GAS strains under different culture conditions were subjected to Northern blot analysis. DNA probes used for detecting the *lacD.1* gene and chloramphenicol cassette were amplified by primers described in **Table 2** and labeled with alkaline phosphatase (AlkPhos Direct Labeling and Detection System, GE Healthcare, Chicago, IL, USA) according to the manual. After hybridization, the membrane was washed and the signal was detected by the Gel Doc XR+ system (BioRad, Hercules, CA, USA).

**Table 2:** Primers used in this study.

Primer	Use	Sequence (5′-3′)^#^	Reference
covR-F-2	Construction	gcggaattctctggtattagttttagacaaagacgc	This study
covR-D53A-R	Construction	ctggtaacattaaggcaagcaggatt	Dalton and Scott, 2004
covR-R-2	Construction	gcggaattcatgacttatttctcacgaat	This study
covR-D53A-F	Construction	aatcctgcttgccttaatgttaccag	Dalton and Scott, 2004
CovR/S-F-3	Construction	gcggatccgcttgcaagggttgtttgatg	This study
CovR/S-R-2	Construction	gcggatccttaagctactctaactctc	This study
A20-lacD.1-F-1	Real-time PCR	tttggcgttgatgtgctaa	Loughman and Caparon, 2006
A20-lacD.1-R-1	Real-time PCR	gacttccccttcagtaaaaccttc	Loughman and Caparon, 2006
lacD.1-F-4	Construction	cggatccgtggaactggagttggcatt	This study
lacD.1-R-2	Construction	cgagctcgcctgactgagctgcttctt	This study
lacD.1-F-3	Construction	cccccgggggtaaaacaaccttatccta	This study
lacD.1-R-3	Construction	cccccgggggcatgtgatacctctctta	This study
lacD.1-F-2	PCR	cgagctcgtggaactggagttggcatt	This study
lacD.1-F	Southern blot	agattcaaacaattatccccatacttatc	This study
lacD.1-R	Southern blot	tgagtactattgctaagccgtttga	This study
rgg-F-4	Construction	cgggatcctgatcggcaaatactgggtta	This study
rgg-R-3	Construction	cgggatccgccctggagctgttgagata	This study
Rgg-F-3	Real-time PCR	tttgaatgccgaaacatagaaggtt	This study
Rgg-R-2	Real-time PCR	ctaataacaccttgaccaaggcaaa	This study
gyrA-F-3	Real-time PCR	cgtcgtttgactggtttgg	This study
gyrA-R-3	Real-time PCR	ggcgtgggttagcgtattta	This study
speB-F-2	Real-time PCR	tgcctacaacagcactttgg	This study
speB-R-2	Real-time PCR	ggtaaagtaggcggacatgc	This study
speB-F-1	Northern blot	gtgtcggtaaagtaggcgga	This study
speB-R-2	Northern blot	ctttggtaaccgttgaagcc	This study
ska-F-1	Real-time PCR	ttgctgacaaagatggttcg	This study
ska-R-1	Real-time PCR	ccctggtctgaaatcgtcat	This study
spy1793-F-2	Real-time PCR	caatccaaaccctctgctgt	This study
spy1793-R-2	Real-time PCR	ccatcaagtggtcgaaggtt	This study


*^#^underline: restriction enzyme site*

### Construction of *covR* Deletion, *covR/covS trans*-Complemented, and Vector Control Strains

Plasmid pMW506, the *covR* gene interrupted by a chloramphenicol cassette via the *Msl*I site on *E. coli* vector pSF152, was provided by Prof. Jiunn Jong Wu. The plasmid pMW506 was electroporated into A20 and AP3 by the method described previously (Chiang-Ni et al., 2008) to interrupt the *covR* gene by a double-cross homologous recombination (designed SW656 and SW934, respectively).

To construct *covR/covS*-complemented strain, the *covR/S* allele (2505 bp, including promoter region) was amplified from wild type A20 strain using primers CovR/S-F-3 and CovR/S-R-2 (**Table 2**) and ligated into the *Bam*HI site of *E. coli*-GAS shuttle vector pTRKL2 to create pCN111. The plasmid pTRKL2 was kindly provided by T. R. Klaenhammer (Department of Food Science, Southeast Dairy Foods Research Center, North Carolina State University, Raleigh, USA). The backbone of pTRKL2 is a pAMß1-derived Gram-positive vector pIL252. The copy number of pTRKL2 is corresponded to pIL252, which is 6-9 copies in a streptococcal and lactococcal host and 30-40 copies in *E. coli* (O’Sullivan and Klaenhammer, 1993). The plasmid pCN111 and vector pTRKL2 were electroporated into AP3 to generate the *covR/S*-complemented strain (SCN121) and vector control strain (SCN127).

### Construction of CovR_D53A_ Mutation, *lacD.1* Deletion, and *rgg* Over-Expression Strains

Phosphorylation site mutation (D53A) in the *covR* gene was generated by overlap PCR with primers covR-F-2, covR-D53A-R, covR-R-2, and covR-D53A-F listed in **Table 2**. The PCR product (1.1 kb) with D53A amino acid substitution was confirmed by sequencing and ligated into temperature-sensitive *E. coli-Lactococcus* shuttle vector pG^+^HOST9 via the *Eco*RI site. The constructed plasmid was transformed into wild type A20 strain and cultured at 30°C. Transformants were transferred to 37°C to force plasmid integration via a single homologous recombination. Finally, transformants in which the plasmid excised from chromosome via a second recombination were selected in the antibiotic-free plate at 30°C. The phosphorylation site mutation was further verified by sequencing and the strain was designed as SCN128.

To construct the *lacD.1* isogenic mutant, pG^+^HOST9 (erythromycin resistant) and *E. coli* vector pSF152 (spectinomycin resistant) were ligated into the *Eco*RI site. The erythromycin cassette of the constructed vector was further removed by *Bam*HI digestion to generate plasmid pCN143. The *lacD.1* gene with upstream (833 bp) and downstream (921 bp) regions was amplified by primers lacD.1-F-4 and lacD.1-R-2 and ligated into pCN143 with the *Bam*HI site. The *lacD.1* gene was removed by inverted PCR with primers lacD.1-F-3 and lacD.1-R-3 (**Table 2**) and replaced by a chloramphenicol cassette from Vector 78 (Tsou et al., 2010) to generate pCN145. The plasmid pCN145 was transformed into A20, AP3, and SCN128 and transformants were selected by chloramphenicol at 30°C. Transformants were transferred to 37°C with chloramphenicol selection to force plasmid integration via a single or double homologous recombination. Transformants with double homologous recombination were screened by antibiotics (spectinomycin sensitive and chloramphenicol resistant) and the deletion of *lacD.1* gene was further confirmed by sequencing. Isogenic *lacD.1* mutants of A20, AP3, and SCN128 were designed as SCN139, SCN140, and SCN141, respectively.

High-copy-number *E. coli-Streptococcus* shuttle vector pDL278 (Chiang-Ni et al., 2012) was employed to construct the *rgg* over-expression strain. The *rgg* gene with its native promoter was amplified by primers rgg-F-4 and rgg-R-3 (1958 bp) and ligated into pDL278 with the *Bam*HI site (designed as pCN138). pCN138 was transformed into *covS* mutant AP3 to generate *rgg* over-expression strain SCN134.

### Skim-Milk Agar Assay and Western Blot Analysis for SpeB

Skim-milk agar was prepared by Columbia agar base (Becton, Dickinson and Company, Sparks, MD, USA) supplemented with 3% skim milk. GAS strains were sub-cultured on the Skim-milk agar and incubated at 5% CO_2_, 37°C incubator for 12-16 h. Casein hydrolysis by GAS strains results in the appearance of a clear zone around bacterial colonies. For Western blot analysis, 30 μl of GAS culture supernatant was subjected to 12% sodium dodecyl sulfate-polyacrylamide gel electrophoresis followed by transfer to the polyvinylidene difluoride membrane (Millipore, Billerica, MA, USA). The membrane was blocked with 5% skim milk in PBST buffer (PBS containing 0.2% of tween-20) at 37°C for 1 h, and SpeB protein was detected by the anti-SpeB antibody (Toxin Technology, Inc., Sarasota, FL, USA) according to the manual. After hybridization, the membrane was washed with PBST and hybridized with the secondary antibody, peroxidase conjugated goat anti-rabbit IgG (1:10,000 dilution; Cell Signaling Technology, Inc., Danvers, MA, USA) at room temperature for 1 h. The blot was developed using Pierce ECL Western Blotting Substrate (Thermo Fisher Scientific Inc., Rockford, IL, USA) and the signal was detected by the Gel Doc XR+ system (BioRad).

### Phos-Tag Western Blot Analysis for CovR Protein

Group A *Streptococcus* strains were cultured in TSBY broth supplemented with or without 20 mM Mg^++^. Bacteria were grown to the stationary phase of growth, and bacterial pellets were washed and resuspended in the Buffer A. Bacterial cells were disrupted by the bead beater (Mini-Beadbeater, BioSpec Products Inc., Bartlesville, OK, USA) and supernatants were collected for further analysis. Phos-tag SDS-PAGE was prepared according to the manual. Briefly, 10 μg of bacterial total protein was mixed with the 4× protein dye and subjected to 10% SDS-PAGE containing 10 μmol/L of Phos-tag (Wako Pure Chemical Industries Ltd, Richmond, VA, USA) and 0.5 μM of MnCl_2_ directly without boiling. Phosphorylated proteins were separated on Phos-tag SDS-PAGE for 120-140 min at 100 V at 4°C. The gel was washed with transfer buffer (39 mM of glycine, 48 mM of Tris, 0.037% of SDS, and 20% of methanol) supplemented with 1 mM of EDTA for 40 min. The gel was washed again by EDTA-free transfer buffer for another 20 min and proteins were transferred onto the PVDF membrane. The membrane was blocking by 5% skim milk in PBST buffer at 37°C for 1 h. Mouse anti-CovR serum (kindly provided by Prof. Jiunn Jong Wu) in 1:5,000 dilutions was used as the primary antibody for hybridization. After hybridization, the primary antibody was washed from the membrane with PBST buffer and hybridized with the secondary antibody, peroxidase conjugated goat anti-mouse IgG (1:10,000 dilution; Millipore, Billerica, MA, USA) at room temperature for 1 h. The blot was developed using Pierce ECL Western Blotting Substrate and the signal was detected by the Gel Doc XR+ system (BioRad).

### Statistical Analysis

Statistical analysis was performed by using the Prism software, version 4 (GraphPad, San Diego, CA, USA). Significant differences in multiple groups were determined using ANOVA. Post-test for AVOVA was analyzed by Tukey’s Honestly Significant Difference Test. A *p*-value <0.05 was taken as significant.

## Results

### CovR Negatively Regulates *speB* Expression in Both Wild Type and *covS* Mutant Strains

**FIGURE 1:**
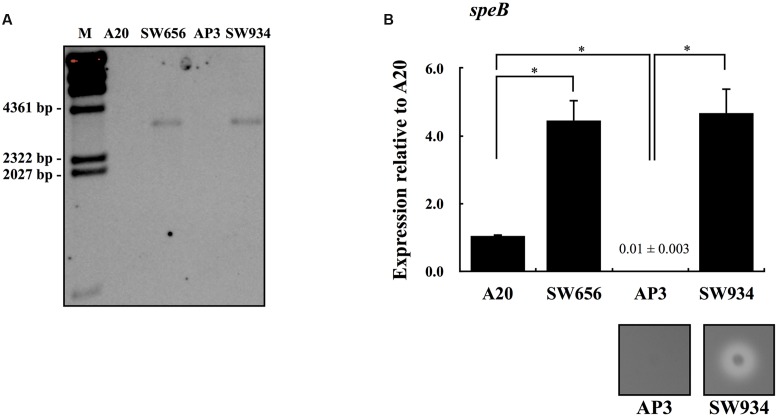
**The expression of *speB* in wild type A20 strain, *covS* mutant AP3, and their *covR* isogenic mutants.** **(A)** Southern blot analysis for A20, AP3, and their *covR* isogenic mutants SW656 and SW934. Genomic DNAs were extracted and digested with *Eco*RV. The interruption of the *covR* gene by a chloramphenicol (Cm) cassette was detected by the Cm probe. M, λ/*Hin*dIII DNA marker. The expression of *speB* in **(B)** A20, SW656, AP3, and SW934 in the stationary phase of growth. Group A *Streptococcus* (GAS) strains were cultured in broths for 7 h and RNAs were extracted for quantitative PCR (qPCR) analysis. Biological replicate experiments were performed using four independent RNA preparations in duplicate. The expression of *speB* was normalized to the *gyrA*. ^∗^*p* < 0.05. The lower panel in **(B)** shows the protease activity of AP3 and SW934. Bacteria were cultured in the 3% skim-milk agar for 12-16 h, and the appearance of a clear zone around bacterial colony is considered protease positive.

Although CovR protein can be autophosphorylated in the absence of CovS, inactivation of CovS results in a dramatically decreased level of phosphorylated CovR (Miller et al., 2001; Dalton and Scott, 2004; Horstmann et al., 2015). However, expression of *speB* is downregulated in *covS* mutants compared with wild type strains (Sumby et al., 2006; Walker et al., 2007; Trevino et al., 2009; Tran-Winkler et al., 2011). These results suggest that CovR acts as a repressor of *speB* expression, even in the absence of sensor kinase CovS. To elucidate the role of CovR in the regulation of *speB* expression in the presence or absence of CovS, isogenic mutants lacking functional *covR* were constructed from wild type A20 (SW656) and *covS* mutant AP3 (SW934) strains. The interruption of the *covR* gene by a chloramphenicol (Cm) cassette in SW656 and SW934 was further confirmed by Southern blot analysis. Results showed that the Cm cassette was only detected in SW656 and SW934 with a size of 3.3 kb, but not in their parental strains (**Figure 1A**). Expression of *speB* in the stationary phase of growth was upregulated in SW656 compared with A20 (**Figure 1B**). In addition, expression of *speB* was also upregulated in SW934 compared with AP3 (**Figure 1B**). Furthermore, SW934, but not AP3, hydrolyzed casein in the skim-milk agar plate (**Figure 1B**, lower panel). These results indicate that CovR acts as a negative regulator of *speB* expression in both wild type and *covS* mutant strains.

### Overexpression of *rgg* in the *covS* Mutant Restores *speB* Expression

**FIGURE 2:**
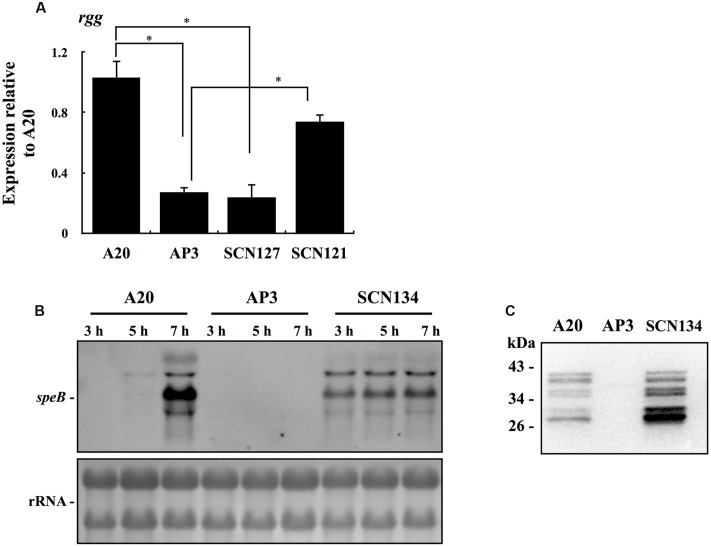
**The expression of *rgg* and *speB* in wild type A20 strain, *covS* mutant AP3, *covR/S-*complemented strain SCN121, vector control strain SCN127, and AP3 *rgg* overexpression strain SCN134.** **(A)** The expression of *rgg* in A20, AP3, SCN127, and SCN121 in the stationary phase of growth. **(B)** The expression of *speB* in A20, AP3, and SCN134 in the early-exponential (3 h), late-exponential (5 h), and stationary phases (7 h) of growth. GAS strains were cultured in TSBY broth to different phases of growth and RNAs were extracted for Northern blot analysis. rRNA (lower panel) is used as the internal loading control. **(C)** The production of SpeB protein in A20, AP3, and SCN134. GAS strains were cultured in TSBY broth to the stationary phase of growth and 30 μl of bacterial culture supernatants were subjected to Western blot analysis with the anti-SpeB antibody.

Phosphorylated CovR protein has a greater affinity for the *speB* promoter DNA than non-phosphorylated CovR (Miller et al., 2001). Therefore, we proposed that CovR does not directly repress the expression of *speB* in the *covS* mutant. Sumby et al. (2006) showed that the expression of SpeB-positive regulator Rgg in the *covS* mutant is significantly decreased compared with that in the wild type strain. In line with the previous observation, we also found that expression of *rgg* was downregulated in the *covS* mutant AP3 compared with that in the wild type A20 strain (**Figure 2A**). In addition, *covR/covS trans*-complemented strain SCN121, but not vector control strain SCN127, restored *rgg* expression in the stationary phase of growth of AP3 (**Figure 2A**). To elucidate the role of *rgg* repression in *speB* expression in the *covS* mutant, *rgg* was overexpressed by the high-copy number vector pDL278 in AP3 (SCN134). Expression of *speB* is repressed in the *covS* mutant; therefore, the value of the fold change in expression cannot be precisely evaluated by a relative quantification PCR method. Therefore, the expression of *speB* in AP3 and SCN134 was analyzed by Northern blotting. Results showed that *speB* transcripts were detected in SCN134 but not in AP3 (**Figure 2B**). Compared to the wild type A20 strain, the expression of *speB* in the stationary phase of growth in SCN134 was only partially restored (7 h of incubation, **Figure 2B**). Expression of *speB* is restricted to the stationary phase of growth (Unnikrishnan et al., 1999; Neely et al., 2003); however, we found that *speB* transcripts can be detected in SCN134 in the early- and late-exponential phases of growth (3 and 5 h of incubation, respectively, **Figure 2B**). SpeB protein in the bacterial culture supernatant was further detected by Western blot. SpeB is secreted as an inactive precursor (42 kDa), which is autocatalytically cleaved into an active protease (28 kDa), and produces at least six intermediates during this process (Doran et al., 1999; Collin and Olsen, 2000; Zimmerlein et al., 2005). Our results show that the mature SpeB protein and its intermediates were detected in the culture supernatant of SCN134, but not in AP3 during the stationary phase of growth (**Figure 2C**). These results indicate that *rgg* repression contributes directly to *speB* inhibition in the *covS* mutant.

### Expression of *rgg* Is Repressed by the Response Regulator CovR in the *covS* Mutant

**FIGURE 3:**
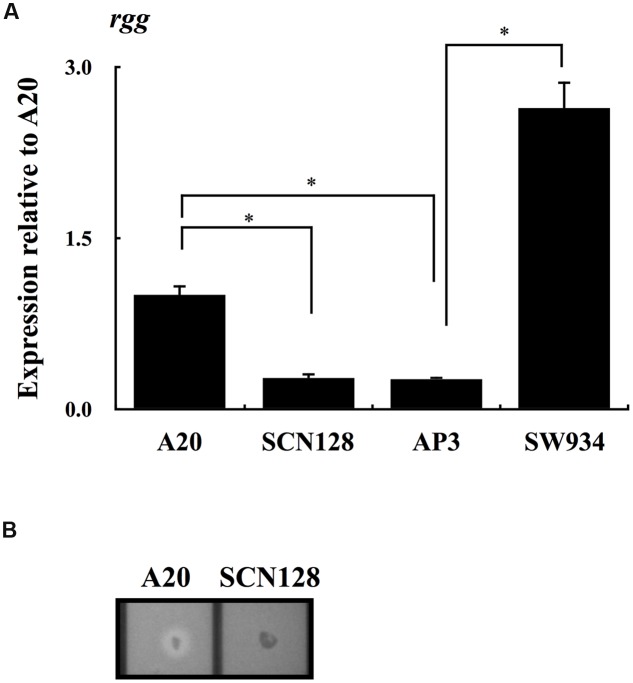
**The expression of *rgg* in A20, A20 CovR_D53A_ mutation strain SCN128, *covS* mutant AP3, AP3 *covR* mutant SW934.** The expression of *rgg* in **(A)** A20, SCN128, AP3, and SW934 in the stationary phase of growth. All qPCR analyses were performed using three independent RNA preparations in duplicate. The expression of *rgg* was normalized to the *gyrA*. ^∗^*p* < 0.05. **(B)** Shows the protease activity of A20 and SCN128. Bacteria were cultured in the 3% skim-milk agar for 12-16 h, and the appearance of a clear zone around the bacterial colony is considered protease positive.

Results from **Figure 2A** show that the expression of *rgg* was repressed in AP3 compared with that in the wild type A20 strain. To elucidate the role of CovR in regulation of *rgg* expression in the absence of CovS, the expression of *rgg* in AP3 and its *covR* isogenic mutant SW934 was analyzed. The results showed that expression of *rgg* during the stationary phase of growth increases significantly in SW934 compared with AP3 (**Figure 3A**), suggesting that CovR negatively regulates *rgg* expression. AP3 is not the *covS* isogenic mutant of A20. Although we considered that only a T nucleotide deletion in the *covS* in AP3 genome is important, the possibility that other unidentified mutations in AP3 contribute to the *rgg* repression cannot be completely excluded. Regulation by CovS is thought to be solely through CovR (Trevino et al., 2009). Therefore, to study the role of CovS inactivation in *rgg* expression, a wild type A20 strain was constructed with the mutation in phospho-aspartic acid residue (D53A) of CovR (SCN128) (Dalton and Scott, 2004). Expression of *rgg* was significantly decreased in SCN128 compared with the wild type A20 strain (**Figure 3A**). In addition, *speB* transcripts and protease activity were not detected in SCN128 (**Figure 3B** and data not shown). These results suggest that the nonphosphorylated CovR protein in the *covS* mutant acts as a transcriptional repressor of *rgg* expression.

### Expression of *rgg* Is Negatively Regulated by Both Phosphorylated and Non-phosphorylated CovR

**FIGURE 4:**
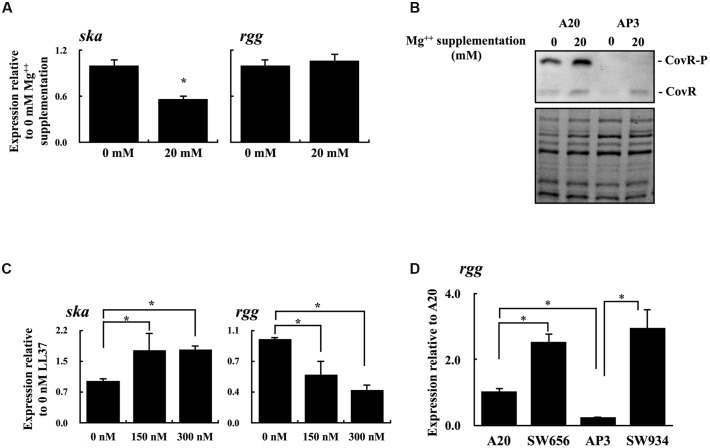
**The phosphorylation level of CovR protein and the expression of *ska* and *rgg* in A20 under Mg^++^ and LL37 stimulation.** **(A)** The expression of *ska* and *rgg* in A20 with or without the high concentration of Mg^++^ stimulation. **(B)** Phos-tag Western blot for detecting the phosphorylated and non-phosphorylated CovR in A20 and AP3, with or without a high concentration of Mg^++^ stimulation. CovR-P indicates the phosphorylated CovR protein; CovR indicates the non-phosphorylated CovR protein. The lower panel shows total protein as a loading control. **(C)** The expression of *ska* and *rgg* in A20 with 0, 150, and 300 nM of LL37 treatments. **(D)** The expression of *rgg* in A20, AP3, and their *covR* isogenic mutants (SW656 and SW934, respectively) in the stationary phase of growth. All qPCR analyses were performed using four independent RNA preparations in duplicate. The expression of *ska* and *rgg* were normalized to the *gyrA*. ^∗^*p* < 0.05.

Results showed in **Figure 3** suggests that the decrease in the level of phosphorylated CovR results in downregulation of *rgg* expression. The CovR/CovS system responds to changes in Mg^++^ concentrations by changing the phosphorylation status of CovR (Tran-Winkler et al., 2011; Horstmann et al., 2015). Therefore, the expression of *rgg* and the CovR-regulated gene *ska* in A20 and AP3 under Mg^++^ stimulation was further analyzed. As expected, the expression of *ska* was decreased significantly with Mg^++^ stimulation in A20 but not in AP3 (**Figure 4A** and data not shown). However, there was no statistical difference in the levels of *rgg* expression between Mg^++^ treated and nontreated A20 or AP3 (**Figure 4A** and data not shown). Horstmann et al. (2015) showed that serotype M1 GAS strains have high levels of phosphorylated CovR in the absence of Mg^++^ stimulation; therefore, differences in the expression levels of *rgg* in A20 with or without Mg^++^ treatment could not be clearly observed. Therefore, CovR phosphorylation levels in A20 and AP3 in the presence with or without Mg^++^ were further analyzed by Phos-tag western blot (Barbieri and Stock, 2008; Horstmann et al., 2015). Results showed that phosphorylated CovR protein was only clearly observed in A20 but not in AP3 (**Figure 4B**). In addition, a slight increase in phosphorylated CovR protein in A20 was found under Mg^++^ stimulation (**Figure 4B**), indicating that A20 has high levels of phosphorylated CovR in the absence of Mg^++^ stimulation. Horstmann et al. (2015) showed that levels of phosphorylated CovR in M1 GAS strain decreases significantly in the presence of antimicrobial peptide LL37. To further elucidate the role of phosphorylation level of CovR in the regulation of *rgg* expression, the wild type A20 strain was treated with LL37 and the expression of *ska* and *rgg* was analyzed. A20 strain was grown to the late-exponential phase of growth and then treated by 150 and 300 nM LL37 for another 3 h. After LL37 treatment, the expression of *ska* increased significantly compared with that in A20 incubated in TSBY broth without supplemented with LL37 (**Figure 4C**). In addition, the expression of *rgg* was decreased in the presence of 150 and 300 nM of LL37 (**Figure 4C**). Furthermore, the role of phosphorylated and non-phosphorylated CovR protein in the regulation of *rgg* expression was further evaluated by *covR* isogenic mutants of A20 and AP3. Expression of *rgg* was significantly increased in SW656 and SW934 compared with the parental strains (**Figure 4D**), indicating that CovR acts as a transcriptional repressor in both wild type and *covS* mutant strains. In addition, these results also suggest that non-phosphorylated CovR can more strongly repress *rgg* expression than phosphorylated CovR.

### Expression of *lacD.1* Is Derepressed in the *covS* Mutant

**FIGURE 5:**
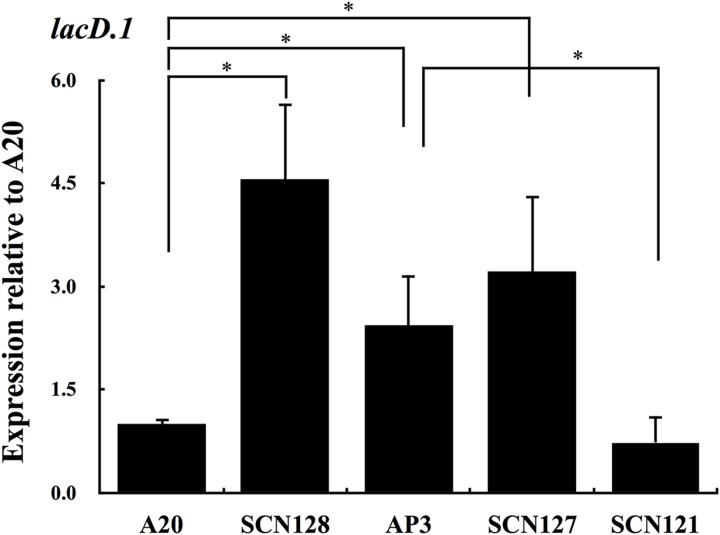
**The expression of *lacD.1* in wild type A20 strain, A20 CovR_D53A_ mutation strain SCN128, *covS* mutant AP3, *covR/S-*complemented strain SCN121, and vector control strain SCN127.** The expression of *lacD.1* in A20, SCN128, AP3, SCN127, and SCN121 in the stationary phase of growth. All qPCR analyses were performed using three independent RNA preparations in duplicate. Expression of the *lacD.1* was normalized to the *gyrA*. ^∗^*p* < 0.05.

Expression of *speB* in SCN134 was only partially restored (**Figure 2B**), suggesting that the regulatory activity of Rgg in SCN134 could still be inhibited. Therefore, the expression of *lacD.1*, a negative regulator of Rgg, was analyzed. The results showed that expression of *lacD.1* was significantly increased in the *covS* mutant AP3 compared with that in the wild type A20 strain (**Figure 5**). In addition, the AP3 *covR/S trans*-complemented strain SCN121, but not the vector control strain SCN127, showed similar *lacD.1* expression levels to A20 in the stationary phase of growth (**Figure 5**). The role of CovS inactivation in *lacD.1* upregulation was further elucidated by SCN128. Compared to the wild type A20 strain, SCN128 showed a significantly increase in *lacD.1* expression. These results indicate that expression of *lacD.1* is upregulated in the *covS* mutant.

### LacD.1 Is Not Involved in *speB* Repression in the *covS* Mutant

**FIGURE 6:**
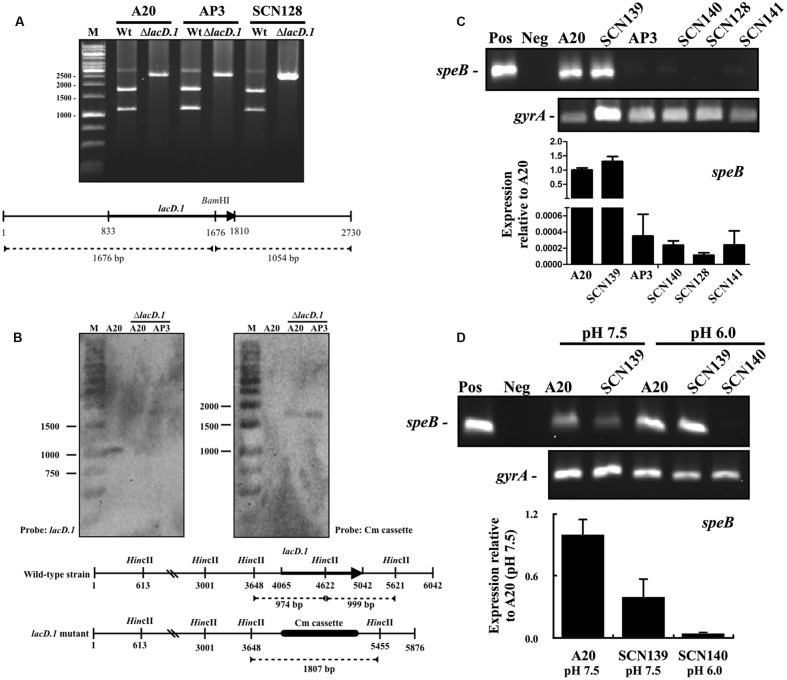
**The expression of *speB* in wild type A20 strain, *covS* mutant AP3, A20 CovR_D53A_ mutation strain SCN128, and their isogenic *lacD.1* mutants.** Deletion of the *lacD.1* gene in *lacD.1* mutants was verified by restriction enzyme digestion and Southern blot analysis. **(A)** The *Bam*HI digestion pattern of the partial *lac.1* operon in wild type strains and their isogenic *lacD.1* mutants. The lower panel shows the location of the *lacD.1* gene, *Bam*HI restriction enzyme sites, and predicted size after *Bam*HI digestion. M, DNA marker. **(B)** Southern blot analysis for A20, A20 *lacD.1* mutant, and AP3 *lacD.1* mutant. Genomic DNAs were digested by *Hin*cII and separated by 1% agarose gel for Southern blot analysis. The lower panel shows the location of the *lacD.1* gene in wild type strains, a Cm cassette in *lacD.1* mutants, *Hin*cII restriction enzyme sites, and the predicted size for *lacD.1* and Cm probe hybridization. M, DNA marker. **(C)** The expression of *speB* in A20, AP3, SCN128, and their isogenic *lacD.1* mutants (SCN139, SCN140, and SCN141, respectively). GAS strains were cultured in TSBY broth to the stationary phase of growth and RNAs were extracted for RT-PCR and qPCR (lower panel) analyses. **(D)** The expression of *speB* in A20, AP3, and their isogenic *lacD.1* mutants at pH 7.5 and pH 6.0 conditions. GAS strains were cultured in neutral and acidic pH broths for 3 h and RNAs were extracted for RT-PCR analysis. The expression of *gyrA* gene is used as the internal control. Pos and Neg: Positive control (genomic DNA as template) and negative control. Lower panel, qPCR analysis for the *speB* expression in A20 and SCN139 under pH 7.5 condition and SCN140 under pH 6.0 condition. Biological replicate experiments were performed using three independent RNA preparations in duplicate. The expression of *speB* was normalized to the *gyrA*.

The increase in *lacD.1* expression may lead to Rgg inactivation in the *covS* mutant AP3 (Loughman and Caparon, 2006). Therefore, the role of LacD.1 in *speB* inhibition in the *covS* mutant was further analyzed. Isogenic *lacD.1* mutant of A20, AP3, and SCN128 were constructed. The deletion of *lacD.1* gene in *lacD.1* mutants was confirmed by sequencing analysis (data not shown). In addition, a 2.7 kb region of the *lac.1* operon of wild type and *lacD.1* mutant strains was amplified by PCR with primers lacD.1-F-2 and lacD.1-R-2 (**Table 2**), and PCR products were further digested with the restriction enzyme *Bam*HI. The *Bam*HI site is located within the *lacD.1* gene; therefore, as expected, only PCR products amplified from the wild type strains were digested by *Bam*HI (**Figure 6A**). The *lacD.1* and *lacD.2* genes have 70% sequence identity; therefore, the *lacD.1* mutants were further verified by Southern blot analysis. Results showed that no *lacD.1* gene was detected in DNA extracted from the *lacD.1* isogenic mutants of A20 and AP3 (**Figure 6B**). In addition, the Cm cassette was only detected in the *lacD.1* mutants (**Figure 6B**). However, deletion of the *lacD.1* gene in AP3 (SCN140) and SCN128 (SCN141) did not restore *speB* expression (**Figure 6C**). Expression of *speB* is induced by acidic pH but inhibited by neutral pH (Loughman and Caparon, 2006; Chiang-Ni et al., 2012). LacD.1 has been shown to play critical roles in the inhibition of *speB* expression under neutral pH culture conditions (Loughman and Caparon, 2006). However, this study found that *speB* expression was inhibited in both the wild type A20 strain and its *lacD.1* isogenic mutant (SCN139) under neutral pH culture conditions (**Figure 6D**). In addition, the expression level of *speB* in SCN140 under acidic condition was still lower than that of A20 and SCN139 under pH 7.5 condition (**Figure 6D**). These results indicate that LacD.1 is not involved in the repression of *speB* expression in the *covS* mutant.

## Discussion

Along with polymorphisms of the *covR/S* genes, mutations in the *rgg* gene are detected more frequently in strains isolated from patients with invasive infection (Ikebe et al., 2010). Friaes et al. (2015) suggested that *covR/S* and *rgg* genes are under stabilizing selection. However, only the deletion of *covS*, not *rgg*, is associated with invasive infections (Friaes et al., 2015). Mutations in *covS* have been shown to inactivate CovR phosphorylation and derepress target genes expression (Horstmann et al., 2015). However, the expression of CovR-regulated gene, *speB*, is repressed in the *covS* mutant. The present study demonstrated that nonphosphorylated CovR in the *emm1*-type A20 strain represses *rgg* expression (**Figures 2** and **3**), and the repression of *rgg* in *covS* mutant directly contributes to inhibiting *speB* expression (**Figure 2**). Furthermore, these results also suggest that the alteration of Rgg regulon expression in the *covS* mutant may contribute to higher invasiveness and provide better fitness in specific niches during infection.

CovR acts primarily as a transcriptional repressor to restrict expression of genes that it regulates directly (Churchward, 2007). The present study shows that CovR acts to repress *rgg* transcription in both wild type and *covS* mutant strains (**Figure 4D**). However, the results showed in **Figures 3A**,**4C** suggest that nonphosphorylated CovR more strongly inhibits *rgg* expression than phosphorylated CovR. The AT-rich CovR-binding sequences (ATTARA) have been proposed and identified in the promoter regions of *hasA*, *covR*, *sagA*, *ska*, and *speB* (Bernish and van de Rijn, 1999; Miller et al., 2001; Federle and Scott, 2002; Gao et al., 2005; Gusa and Scott, 2005; Gusa et al., 2006; Churchward et al., 2009). A CovR-binding sequence can be found in the coding region of the *rgg* gene^2^ (98-113; Virtual Footprint); however, this sequence cannot be identified in the *rgg* promoter region. In addition, phosphorylation of CovR increases its DNA-binding affinity and specificity for all promoter DNA fragments described above. Based on these results, we suggest that CovR may not bind to the *rgg* promoter and repress *rgg* transcription directly in the *covS* mutant. However, the interactions between phosphorylated and nonphosphorylated CovR proteins and the *rgg* gene are still uncertain and should be further studied.

The genome of GAS contains two loci (Lac.1 and Lac.2) encoding putative components of the tagatose 6-phosphate pathway (Ferretti et al., 2001; Loughman and Caparon, 2007). A gene encoding tagatose-1,6-bisphosate aldolase is present at both loci (*lacD.1* and *lacD.2*); however, only LacD.2 contributes to utilize carbohydrate (Loughman and Caparon, 2007). LacD.1 has been shown to interact with Rgg to prevent Rgg-mediated gene expression in *emm14*- and *emm49*-type strains (Loughman and Caparon, 2006; Anbalagan et al., 2012). In addition, Rgg is a transcriptional regulator of genes associated with stress responses, metabolism, and virulence in *emm3-* and *emm49*-type strains (Chaussee, 2002; Chaussee et al., 2004; Pulliainen et al., 2008; Ikebe et al., 2010). However, among different *emm*-type strains, only the expression of *speB* and adjacent gene *spy2040* are negatively regulated by Rgg (Dmitriev et al., 2008). Therefore, the expression of *speB* is a reliable marker to evaluate the interaction between LacD.1 and Rgg. The present study shows that the deletion of *lacD.1* in AP3 did not restore *speB* expression (**Figure 6C**). Overexpression of *rgg* in AP3 activated *speB* expression in the early-exponential phase of growth (**Figure 2B**). In addition, *speB* expression in the wild type A20 strain and its *lacD.1* isogenic mutant was still repressed under neutral culture conditions (**Figure 6D**). These results suggest two possible mechanisms in this A20 strain: LacD.1 does not interact with Rgg or LacD.1 binds to Rgg but does not inhibit Rgg-mediated *speB* expression. Both the AP3 strain used in this study and MGAS5005 are *emm1*-type strains and have an identical T nucleotide deletion in the *covS* gene. Dmitriev et al. (2008) showed that expression of *speB* and *spy2040* are downregulated and *spy1793* is upregulated in an MGAS5005 *rgg* isogenic mutant, indicating that Rgg negatively regulates *spy1793* expression. To further elucidate the role of LacD.1 in the regulation of Rgg activity, the expression of *spy1793* was analyzed by qPCR. However, although the expression of *spy1793* was upregulated in the *covS* mutant AP3 compared with that in wild type A20 strain (expression relative to A20: 2.67 ± 0.8), inactivation of *rgg* in A20 (data not shown) showed to repress *spy1793* expression (expression relative to A20: 0.34 ± 0.09), suggesting that *spy1793* is regulated by multiple regulators in the *covS* mutant. These results suggest that *spy1793* is not a suitable marker to evaluate the Rgg activity; in addition, more efforts are needed to clarify the interactions between LacD.1 and Rgg among different GAS strains.

Trevino et al. (2009) showed that CovS activates CovR to repress one group of genes, while it simultaneously inhibits the ability of CovR to repress the second group of genes (e.g., *speB*). Phosphorylated CovR has greater DNA-binding affinity and specificity for target gene promoters than that of nonphosphorylated CovR. Therefore, it is unlikely that CovS phosphorylates CovR leading to phosphorylated CovR failing to bind to the *speB* promoter. The present study shows that the inactivation of CovS results in a decrease in the level of CovR phosphorylation, and nonphosphorylated CovR mediates *rgg* repression (**Figures 3** and **4**). Rgg is essential for triggering *speB* expression (Neely et al., 2003). Therefore, although non-phosphorylated CovR has a lower binding activity to the *speB* promoter (Miller et al., 2001), the *covS* mutant still failed to express *speB* due to the downregulation of *rgg*. This study did not reveal the molecular mechanism of the CovR-Rgg interaction; however, these results suggest the importance of Rgg in gene regulation in the invasive *covS* mutant. Further explorations of the interactions between these regulatory systems in the invasive *covS* mutant would shed light on the disease pathogenesis of invasive GAS infections.

## Author Contributions

The conception or design of the study: CC-N, C-HC, and J-JW. The acquisition, analysis, or interpretation of the data: T-PC and CC-N. Writing of the manuscript: CC-N.

## Conflict of Interest Statement

The authors declare that the research was conducted in the absence of any commercial or financial relationships that could be construed as a potential conflict of interest.

## Footnotes

1 http://frodo.wi.mit.edu

2 http://www.prodoric.de/vfp/
